# Drinking status but not acute alcohol consumption influences delay discounting

**DOI:** 10.1002/hup.2617

**Published:** 2017-08-09

**Authors:** Sally Adams, Angela S. Attwood, Marcus R. Munafò

**Affiliations:** ^1^ UK Centre for Tobacco and Alcohol Studies and Department of Psychology University of Bath Bath; ^2^ UK Centre for Tobacco and Alcohol Studies and School of Experimental Psychology University of Bristol Bristol; ^3^ MRC Integrative Epidemiology Unit (IEU) at the University of Bristol Bristol

**Keywords:** alcohol, delay discounting, impulsivity

## Abstract

**Objective:**

The aim of this study was to investigate the following: (a) the effects of acute alcohol on delay discounting; (b) the effects of drinking status on delayed discounting; and (c) whether these effects differ according to reward type (alcohol vs. money).

**Methods:**

Heavy and light social alcohol users (*n* = 96) were randomized to receive either an acute dose of alcohol at 0.4 or 0.6 g/kg or placebo in a between‐subjects, double‐blind design. Delay discounting of alcohol and monetary rewards was measured using a hyperbolic model, with higher scores indicative of greater delay discounting.

**Results:**

ANOVA of discount scores indicated a main effect of reward type, where all participants had higher discount scores for alcohol versus money rewards. A main effect of drinking status was also observed, where heavier drinkers had higher discount scores compared with lighter drinkers. We did not observe a main effect of acute alcohol use on delay discounting or the hypothesized interactions between acute alcohol use and drinking status with reward type.

**Conclusions:**

Our data suggest that heavier drinkers discount the value of delayed rewards more steeply than lighter drinkers. Delay discounting may therefore be a promising marker of heavy alcohol consumption in social drinkers.

## 1 INTRODUCTION

Delay discounting is defined as the impulsive choice of smaller, immediate rewards over larger, delayed rewards (Rachlin & Green, 1972). Delay discounting tasks assess the extent to which decision‐making is insensitive to longer‐term consequences and rewards, with participants making a choice between receiving a smaller immediate reward or a larger delayed reward. In the case of alcohol, impulsive choice may reflect choosing a lesser, immediate reward (e.g., intoxication and euphoria), over a larger, delayed reward (e.g., good health). Laboratory studies examining the acute effects of alcohol on delay discounting have produced mixed findings. Studies in both human and rodents report that moderate to high doses of alcohol (0.25–0.8 g/kg) have no effect on delay discounting (Richards, Zhang, Mitchell, & de Wit, 1999, Wilhelm & Mitchell, 2012, Bidwell et al., 2013, Wray, Simons, & Maisto, 2015). In contrast, other studies have shown a trend for reduced (Ortner, MacDonald, & Olmstead, 2003) and increased delay discounting following high dose of alcohol (0.7–0.8 g/kg; Reynolds, Richards, & de Wit, 2006).

Another line of research has indicated that alcohol dependent and heavy social drinkers discount the value of monetary rewards more steeply than lighter drinkers (Vuchinich & Simpson, 1998, Petry, 2001a, Mitchell, Fields, D'Esposito, & Boettiger, 2005, Field, Christiansen, Cole, & Goudie, 2007). Additionally, alcohol dependent and social drinkers discount alcohol rewards more steeply than monetary rewards (Petry, 2001b; Odum & Rainaud, 2003). Steeper delay discounting for drug‐related rewards has been demonstrated across a range of substances (e.g., marijuana, cigarettes, and alcohol; Baker, Johnson, & Bickel, 2003, Mitchell, 2004, Estle, Green, Myerson, & Holt, 2007, Johnson et al., 2010). A preference for smaller immediate, drug‐related rewards compared with monetary rewards has two possible theoretical explanations (Odum & Rainaud, 2003). Firstly, a preference for immediate drug rewards may reflect the perishable nature of drugs and the longer lasting appeal of money (e.g., alcohol may be less attractive as a long‐term reward, compared with money). Secondly, drug rewards are primary reinforcers that have a direct effect on behavior (e.g., intoxication, relaxation), making them valuable as immediate rewards. In contrast, monetary rewards are reinforcers that must be exchanged for other goods in order to impact behavior.

To our knowledge, no studies have examined the effects of acute alcohol use and heaviness of drinking on delay discounting of alcohol versus monetary rewards. Such a study has the potential to inform a growing body of research investigating impulsivity as a potential marker for heavy alcohol use. In this study, we examined the influence of heaviness of drinking, alcohol intoxication, and reward type on delay discounting. We examined the effects of two doses of alcohol on delay discounting of alcohol and monetary rewards in light and heavy social drinkers. Both moderate (0.4 g/kg) and high (0.6 g/kg) doses of alcohol were included to assess the extent to which delay discounting of rewards may be differentially sensitive to different priming doses. Additionally, light and heavy drinkers were included to examine the influence of heaviness of drinking on delay discounting. Delay discounting was assessed with a hypothetical, question‐based measure, given that an experiential paradigm with actual alcohol rewards in this study (e.g., 10 pints of beer or 10 glasses of wine in one single session) would have been unethical (Field et al., 2007). We hypothesized that participants primed with an acute dose of alcohol compared with those given a placebo drink would exhibit increased delay discounting of alcohol‐related rewards, relative to monetary rewards. Additionally, we hypothesized that heavier social drinkers compared to lighter drinkers would exhibit increased delay discounting of alcohol‐related rewards, relative to monetary rewards.

## 2 METHODS

### 2.1 Design

The study employed a double‐blind, placebo‐controlled design, comprising two between‐subjects factors of challenge condition (0.0, 0.4, 0.6 g/kg alcohol) and drinking status (light drinkers, heavy drinkers) and a within‐subjects factor of reward type (alcohol, money).

### 2.2 Participants

Social drinkers were recruited from students and staff at the University of Bristol. Participants were assigned to drinking status groups based upon number of alcohol units consumed per week and according to the UK Department of Health recommended weekly guidelines at the time of testing (2010). Light drinkers were defined as individuals who consume ≥l0 and ≤20 units of alcohol per week for males and ≥5 and ≤14 units of alcohol per week for females. Heavy drinkers were defined as individuals who consume ≥21 and ≤50 units per week for males and ≥15 and ≤35 units per week for females (where 1 unit is equivalent to 8 g of ethanol). Participants received £7 each for participation. The study was approved by the Faculty of Science Research Ethics Committee at the University of Bristol.

### 2.3 Materials

The delay discounting procedure was an experimenter‐delivered task adapted from Moore and Cusens (2010). This task provides an ordinal index of participants' discount function using 12‐choice items. For each item, participants were asked “Would you prefer £10 now or £a in 3 months time?”, where only £a varied and items were arranged so that responses would titrate to a participant's approximate 3‐month hyperbolic discount rate (i.e., value = a/[1 + kD], where a is the immediate value of the reward, D is the delay duration, and k is the rate of discount) expressed along an ordinal scale. The main outcome was a calculated discount score. The lowest possible score was “1 for a 3‐month discount rate of 25% and the highest was 13 for a 3‐month discount rate of 99.9%” (Moore & Cusens, 2010, page 2). Higher discount scores are indicative of greater discounting of delayed rewards. All rewards were hypothetical, but participants were instructed to make their choices as if they were going to actually receive the rewards that they selected. Participants were instructed that one unit of alcohol was equal to £1, which was consistent with the price per unit at the time of testing. Alcohol rewards were matched to roughly equivalent monetary rewards; £20 was equivalent to 10 pints of beer or 10 (175 ml) glasses of wine. We fixed the maximum amount of alcohol offered at an amount that could be plausibly consumed in one single drinking session (e.g., 10 pints of beer or 10,175 ml glasses of wine).

Questionnaire measures included self‐report measures of drinking behavior (Alcohol Use Disorders Identification Test; Bohn, Babor, & Kranzler, 1995), impulsivity (Barratt Impulsivity Scale; Patton, Stanford, & Barratt, 1995), sensation seeking (Impulsive Sensation Seeking; Zuckerman, Kuhlman, Joireman, Teta, & Kraft, 1993), mood (Profile of Mood States [POMS]; McNair, Lorr, & Droppleman, 1992), and craving (visual analogue scale [VAS]). The VAS comprised five items “I would like to consume an alcoholic beverage”, “A drink would be very satisfying,” “The thought of consuming an alcoholic beverage is appealing,” “I need to have a drink,” and “I do not want to consume an alcoholic beverage.” Each item was rated on an 80‐mm scale from “*Not at all*” to “*Extremely*.”

### 2.4 Procedure

All participants were tested between noon and 6 pm in a laboratory in the School of Experimental Psychology at the University of Bristol. On the test day, after providing informed consent, all participants completed a screening process to exclude current use of medication and illicit substances, family history of alcoholism, and recent alcohol consumption (i.e., within 12 hr of test session, verified by exhaled breath alcohol). Participants were required to regularly consume wine or beer as these were the alcohol‐related rewards offered in the delay discounting tasks and (units per week) were recorded to establish allocation to drinking status group (light drinkers, heavy drinkers). Weight was recorded for drink preparation. Following the completion of baseline measures (Alcohol Use Disorders Identification Test, Barratt Impulsivity Scale, Impulsive Sensation Seeking, POMS, and VAS), participants were given 10 min to consume the drink.

Participants were randomly allocated to receive either an alcoholic (0.4, 0.6 g/kg) or a placebo drink. For a 60‐kg adult, the 0.4‐g/kg dose is approximately equivalent to 2 units of alcohol and the 0.6‐g/kg dose to 3 units. The alcohol administration procedure was identical to that described previously (Adams, Ataya, Attwood, & Munafo, 2013). Following drink consumption, participants completed the first awareness check to determine whether they were aware if they had received alcohol or placebo. Awareness of the alcohol content of the challenge condition was determined by asking participants whether they believed that their drink contained alcohol or not.

Next participants were given 15‐min absorption time, to ensure all participants had an equivalent period between challenge administration and the delay discounting task. During this period, participants completed postchallenge measures (POMS, VAS). At the end of this time, participants completed the delay discounting task with money and alcohol reward order counterbalanced across participants. The task lasted approximately 5 min. Following the task, participants completed the POMS and VAS measures again and a second awareness check.

On completion of the study procedure, participants were informed of their drink condition (verified by exhaled breath alcohol) and were reimbursed and provided with a full debrief.

### 2.5 Statistical analysis

All analyses included two between‐subject factors of challenge condition (0.0, 0.4, 0.6 g/kg) and drinking status (light drinkers, heavy drinkers). For the delay discounting task, a mixed‐model ANOVA of discount scores was conducted including a within‐subjects factor of reward type (alcohol, money). Skewness tests for normality indicated that discount data were nonnormal but could be corrected by a log transformation, using log10. For mood and alcohol craving data, mixed‐model ANOVAs of POMS and VAS scores were conducted with an additional within‐subjects factor of time (baseline, postchallenge, posttasks). Bivariate correlations were conducted of delay discounting scores (alcohol, money) with VAS craving scores (baseline, postchallenge, posttasks). A post hoc sensitivity analysis indicated that the study had 80% statistical power at an alpha level of 5% to detect an effect size of *f* = 0.16 for the interaction effect of challenge condition (0.0, 0.4, and 0.6 g/kg alcohol) on reward type (alcohol, money). The data that form the basis of the results presented here are available from the data.bris Research Data Repository (http:/data.bris.ac.uk/data/) doi: 10.5523/bris.j2fzlhxc2or234cki8igwtzr.

## 3 RESULTS

### 3.1 Characteristics of participants

Participants (*n* = 96; 51% male) were, on average, aged 24 years (*SD* = 4, range 18–39). Table 1 shows characteristics of light and heavy drinking participants allocated to challenge conditions. Allocation of light and heavy drinking participants was equally spilt across 0.0 and 0.6 g/kg conditions, with one participant in the 0.4 g/kg miscategorised as a heavy drinker at data collection, which was corrected during data analysis. Exhaled breath alcohol level (BrAL) was 0.00 μg/L at baseline for all participants. At the end of testing, average BrAL was 0.00 μg/L in the placebo (0.00 g/kg alcohol) condition, 0.15 μg/L in the 0.4 g/kg alcohol condition, and 0.25 μg/L in the 0.6 g/kg alcohol condition. At the end of testing, BrAL did not differ between light and heavy drinkers (*p =* .32, η_2_ = 0.01).

**Table 1:** Characteristics of participants

	Alcohol (0.0 g/kg) *N* = 32		Alcohol (0.4 g/kg) *N* = 32		Alcohol (0.6 g/kg) *N* = 32	
	Light *n* = 16	Heavy *n* = 16	Light *n* = 17	Heavy *n* = 15	Light *n* = 16	Heavy *n* = 16
Age (years)	23 (4)	24 (4)	22 (4)	26 (5)	24 (5)	22 (4)
Alcohol (units/week)	11 (5)	26 (11)	12 (4)	25 (6)	12 (5)	25 (9)
Weight (kg)	68 (14)	69 (11)	65 (8)	68 (10)	69 (12)	68 (8)
BIS	78 (5)	78 (5)	80 (7)	78 (7)	81 (5)	78 (6)
ImpSS	11 (3)	14 (3)	12 (3)	12 (3)	11 (3)	12 (3)
AUDIT	11 (5)	13 (5)	10 (4)	15 (4)	10 (5)	14 (6)
Delay score (alcohol)	4 (3)	3 (3)	5 (4)	6 (3)	4 (3)	5 (3)
Delay score (money)	2 (1)	3 (2)	3 (2)	3 (2)	2 (2)	3 (2)

*Note.* Values are expressed as mean (standard deviation). Delay discounting data are untransformed.

*Note.* AUDIT = Alcohol Use Disorders Identification Test; BIS = Barratt Impulsivity Scale; ImpSS = Impulsive Sensation Seeking.

### 3.2 Delay discounting task

**Figure 1:**
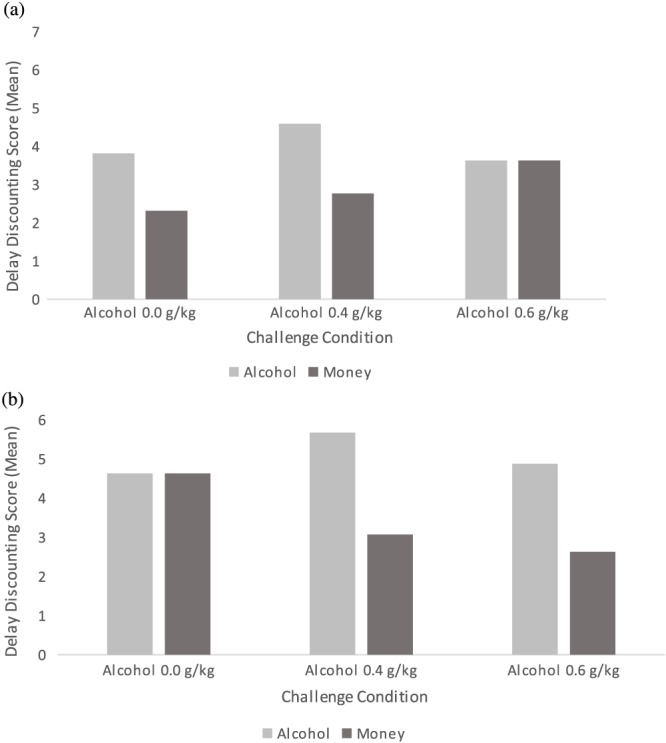
Delay discounting scores by drinking status groups and challenge condition. Discounting scores for alcohol versus money rewards by challenge condition for (a) Lighter drinkers and (b) Heavier drinkers. Values are mean ± SE. Larger values indicate greater discounting of delayed reward. Data are untransformed

A mixed‐model ANOVA of transformed discount scores indicated a main effect of reward type (*F* [1, 90] = 23.94, *p <* .001, η_2_ = 0.21), reflecting higher overall discount scores for alcohol (*M* = 0.5, *SD* = 0.3) compared with money (*M* = 0.3, *SD* = 0.2) rewards. There was also evidence of a main effect of drinking status (*F* [1, 90] = 4.85, *p =* .030, η_2_ = 0.05), with higher discount scores among heavier (*M* = 0.5, *SD* = 0.3) compared with lighter (*M* = 0.4, *SD* = 0.3) drinkers. These data are represented graphically in Figure 1. There was no clear evidence of any other main effects or interactions (*p*s > .20).

### 3.3 Correlations

Pearson correlation coefficients of money and alcohol discount scores with alcohol craving at baseline, postchallenge, and posttasks did not indicate any clear evidence of association between delay discounting and alcohol craving (*r*s < .06, *p*s > .55).

### 3.4 Awareness check

Awareness checks were performed immediately following drink consumption (time 1) and at the end of testing (time 2). At time 1, 47% of participants in the 0.00 g/kg condition, 97% of participants in the 0.4 g/kg condition, and 100% of participants in the 0.6 g/kg condition reported that their drink contained alcohol. At time 2, these figures were 35% in the 0.0 g/kg condition, 97% in the 0.4 g/kg condition, and 100% in the 0.6 g/kg condition.

### 3.5 Craving and mood

A mixed‐model ANOVA of craving scores indicated evidence of a main effect of time (*F* [1, 180] = 4.08, *p =* .021, η_2_ = 0.04), reflecting a linear increase in craving from baseline (*M* = 16, *SD* = 7, *Range* = 1–33) to postchallenge (*M* = 18, *SD* = 13, *Range* = 1–98) to posttasks (*M* = 18, *SD* = 9, *Range* = 1–49). There was no clear evidence of any other main effects or interactions (*p*s > .14).

A mixed‐model ANOVA of mood scores indicated a main effect of time for depression, fatigue, tension, and vigor (*p*s < .01), such that depression, tension, and fatigue decreased linearly across time, while vigor increased from baseline to postchallenge but decreased from postchallenge to posttasks. The main effect of depression was qualified by an interaction between time × challenge condition. Simple effects analyses of time were conducted for participants in the 0.0, 0.4, and 0.6 g/kg conditions separately. There was evidence of a main effect of time for participants in 0.6 g/kg condition only (*F* [2, 56] = 13.77, *p <* .001, η_2_ = 0.33), such that there was a linear decrease in depression from baseline to postchallenge to posttasks. An interaction between drinking status × challenge condition was observed for confusion (*F* [2, 84] = 2.82, *p =* .065, η_2_ = 0.06). Simple effects analyses of time were conducted for participants in the 0.0, 0.4, and 0.6 g/kg conditions separately. There was evidence of a main effect of drinking status for participants in 0.6 g/kg condition only (*F* [1, 27] = 6.87, *p =* .014, η_2_ = 0.20), such that heavier drinkers had higher confusion scores, compared to lighter drinkers. There was no evidence for any other main effects or interactions (*p*s > .11).

## 4 DISCUSSION

Our data suggest that heavier drinkers show greater discounting of delayed rewards relative to lighter drinkers, irrespective of reward type. Additionally, we observed a main effect of reward type, such that all drinkers (in the range examined) showed greater impulsive decision‐making towards alcohol rewards compared with money rewards. Contrary to our hypotheses, our data did not indicate any interaction effects of acute alcohol consumption or drinking status on delayed discounting of alcohol versus money rewards.

Consistent with previous research (Vuchinich & Simpson, 1998, Petry, 2001a, Field et al., 2007), we observed that heavier social drinkers showed steeper delay discounting than lighter drinkers, indicative of greater impulsive decision‐making. However, we did not observe that steeper delay discounting of alcohol versus money rewards was specific to heavier drinking participants, in contrast to previous research (Petry 2001). This discrepancy may reflect differences in the types of drinkers examined, where Petry (2001b) studied alcohol‐dependent drinkers. Our delay discounting data show substantial variation in discounting response in heavier social drinkers, suggesting our sample included a wide range of heavy drinkers (e.g., heavy occasional use to problem drinking). Our results add to a growing body of research indicating that heavier drinkers have a general difficulty in delaying gratification, which may reflect an underlying propensity for greater impulsivity. These findings have implications for reducing alcohol intake by increasing sensitivity to longer‐term rewards (e.g., improved health, social benefits) and the consequences of heavy alcohol use (e.g., the monetary cost of alcohol, the effects of heavy alcohol use on health). Looking ahead, recent research has demonstrated the potential of interventions targeted at reducing impulsive decision‐making in smokers and stimulant drug‐dependent individuals (Bickel, Yi, Landes, Hill, & Baxter, 2011, Hofmeyr, Ainslie, Charlton, & Ross, 2011).

Our findings also suggest that social drinkers (in the range examined) show greater discounting of alcohol versus money rewards. This finding is consistent with previous research (Odum & Rainaud, 2003), suggesting that delay discounting is more pronounced when making a decision concerning alcohol versus money rewards. Our results therefore add to a growing body of research demonstrating steeper discounting of alcohol versus money rewards. Steeper discounting of alcohol rewards may reflect a general process associated with consumable rewards (i.e., alcohol is more valued as an immediate reward due to the fact it can be consumed immediately, whereas money must be exchanged in order to obtain a reward). Additionally, money may be more attractive as a delayed reward, with no expiry.

Consistent with previous research (Richards et al., 1999, Bidwell et al., 2013, Wray et al., 2015), our results suggest that moderate–high doses of alcohol (in the 0.4 to 0.6 g/kg range) do not influence delay discounting of money rewards. Our study is also the first to examine the effects of acute alcohol on discounting of alcohol versus money rewards, indicating no influence of alcohol intoxication on impulsive decision‐making towards alcohol or money rewards. These findings are in contrast to anecdotal reports (Graham, 1980) and previous research (Reynolds et al., 2006), which suggests that acute alcohol increases delay discounting. In addition, our results contradict previous reports of greater discounting of drug versus money rewards during drug intoxication (Giordano et al., 2002, Mitchell, 2004). However, these conclusions are only valid if we accept that delay discounting tasks themselves are sensitive to drug‐induced state changes. Reynolds et al. (2006) suggest that experiential delay discounting tasks are more sensitive to the effects of state changes in impulsivity than question‐based measures, such as the one used in this study. Nevertheless, a recent study (Wray et al., 2015) using an experiential discounting task failed to show an effect of acute alcohol use on delay discounting. Future work should seek to make a direct comparison of different delay discounting tasks on state changes in impulsive decision‐making. Additionally, future work should also examine the reliability and validity of hypothetical measures of delay discounting. Inconsistent findings on the acute effects of alcohol on impulsive behavior reflect the need for replication studies, to establish the role of delay discounting in alcohol‐related state changes in impulsive behavior.

Limitations of our study include the use of hypothetical alcohol‐related and money rewards. As we have noted, some researchers have indicated that hypothetical tasks may be less sensitive to acute changes in discounting (Reynolds et al., 2006). However, consistent with previous research, the provision of actual alcohol rewards in this study would have been unethical, given the amount of drinks offered in a single session (Field et al., 2007). Additionally, our between‐subjects design did not enable collection of baseline information regarding the socioeconomic status of participants. A baseline assessment of socioeconomic status would have enabled us to control for any group differences in value of money rewards. A further limitation of our between‐subjects design was the inability to control for individual differences in impulsive behavior. However, this design was selected to limit participants establishing a stable pattern of responding across time on the delay discounting task (Ortner et al., 2003).

## 5 CONCLUSION

In conclusion, our data indicate that heavier drinkers discount the value of all delayed rewards more steeply than lighter drinkers. This finding suggests that impulsive decision‐making is influenced by individual differences in drinking patterns that is irrespective of reward type. Additionally, we observed that all drinkers in the range examined showed greater delay discounting of alcohol rewards, which may reflect the nature of alcohol as a consumable reward. Our data did not suggest any effects of acute alcohol on delay discounting; however, further research is required to establish how sensitive delay discounting tasks are to state changes in alcohol consumption and to determine the reliability and validity of hypothetical measures of delay discounting. This study adds to a body of research suggesting that impulsive delay discounting is a promising marker of heavy alcohol consumption in social drinkers. Additionally, our data indicate a need for research to explore the mechanism underlying the general trend for steeper discounting of drug versus money rewards observed here and in previous studies.

## CONFLICT OF INTERESTS

Sally Adams, Angela Attwood, and Marcus Munafò are members of the UK Centre for Tobacco and Alcohol Studies, a UKCRC Public Health Research: Centre of Excellence. All other authors declare that they have no conflicts of interest.

## AUTHOR CONTRIBUTIONS

S. A., A. S. A., and M. R. M. designed the study and wrote the protocol. S. A. undertook the statistical analysis and wrote the first draft of the manuscript. All authors contributed to and have approved the final manuscript.
